# A Qualitative Study of Georgian Youth Who Are on the Street or Institutionalized

**DOI:** 10.1155/2012/921604

**Published:** 2012-11-22

**Authors:** Laura K. Murray, Namrita S. Singh, Pamela J. Surkan, Katherine Semrau, Judy Bass, Paul Bolton

**Affiliations:** ^1^Health Systems Program, Department of International Health, Johns Hopkins Bloomberg School of Public Health, 615 N. Wolfe Street, Baltimore, MD 21205-2179, USA; ^2^Social and Behavioral Interventions Program, Department of International Health, Johns Hopkins Bloomberg School of Public Health, 615 N. Wolfe Street, Baltimore, MD 21205-2179, USA; ^3^Department of International Health, Center for Global Health and Development, Boston University School of Public Health, 801 Massachusetts Avenue, 3rd Floor, Boston, MA 02118, USA; ^4^Department of Mental Health, Johns Hopkins Bloomberg School of Public Health, Baltimore, MD 21295, USA

## Abstract

Street children, or children who live and/or spend time on the streets, are a vulnerable group of considerable concern to the global public health community. This paper describes the results of two linked qualitative studies conducted with children living or spending time on the street and in orphanages in and around urban areas in the Republic of Georgia between 2005 and 2006. The studies examined perceived causes of children going to the street, as well as indicators of healthy functioning and psychosocial problems among these children. Results on causes indicated a range of “push” factors leading children to the street and “pull” factors that keep children living on the street. Findings also showed a range of internalizing and externalizing mental health symptoms among children on the street and within orphanages. Some differences in responses were found between children living on the street and in institutions. It is important to understand the perspectives of these vulnerable populations to guide decisions on appropriate interventions that address their primary problems.

## 1. Introduction

Children who live and work on the streets are of significant global public health concern. As a marginalized population, “street children” are at risk of exploitation, health and development problems, including HIV/AIDS, psychosocial problems, drug use, crime, and prostitution [1–4]. Youth who spend time on the street are at risk for increased aggression, experiencing hopelessness, using drugs, and/or becoming sex workers [2, 3, 5–7]. The prevalence of sexually transmitted diseases and drug use has been found to be higher among street children as compared to children living at home [8, 9]. In addition, street children are a medically under-served group, often suffering from malnutrition, as well as developmental and mental health problems [10, 11].

 The term “street children” has been debated [4, 12] but generally includes two separate categories: (1) “children on the street”—children who spend time on the street but usually return to their families at the end of the day and (2) “children of the street”—children who work and live on the streets [13]. Although a number of factors related to family disruption and lack of resources has been strongly associated with children living on the street, the majority of children faced with these problems still remain within the home [14]. It is thus imperative to understand from a public health perspective what brings children to the street, as well as what may keep them there.

Little is known about the population in the former Soviet republics, including the Republic of Georgia, despite the fact that UNICEF (2005) has estimated that 2,500 Georgian children prostitute themselves or beg on the street. Most research on street children has focused on populations in Latin America and Africa, yet Georgia is a uniquely challenging setting within which it is important to consider the health needs of vulnerable children. Since the breakup of the Soviet Union in 1991, Georgia has seen the collapse of basic social services, political and economic instability, armed conflict, and mass displacement of citizens [15–19]. These challenges have contributed to the numbers of children who live on the street, spend large amounts of time on the street, and/or are raised in orphanages in Georgia [3, 29]; these challenges are also linked with poorer mental health outcomes for these children.

 Georgia also faces particular challenges with the organization and delivery of services and care. Health and social services systems in Georgia remain influenced by the structures and institutions of the past, although the country is engaged in a series of health care reforms and is moving towards new systems of care. Presently, Georgia's mental health care system continues to be heavily reliant upon institutions for in-patient psychiatric care of severe cases of mental illness [20]. A lack of available and accessible community-based mental health services means that individuals with mild to moderate forms of mental illness or psychosocial problems and dysfunction are often not effectively treated or helped. Georgia lacks a cohesive and comprehensive community mental health care system. For example, nongovernmental organizations (NGOs) and other international organizations providing social work and mental health care services work at the community level [20], but their services are limited in scope, target population, and resources, and are often short-term. The mental health care system is also underdeveloped in terms of human resources, financial resources, and regular access to newer classes of psychotropic drugs. There are insufficient numbers of skilled mental health care providers at all levels (e.g., psychiatric nurses, psychologists, and psychiatrists), particularly for children and adolescents, as well as inadequate paraprofessional or community health worker resources and continuing medical education for primary care providers [21]. Vulnerable children in Georgia therefore lack adequate access to outpatient services, particularly for more moderate forms of illness and dysfunction, as well as continuity of care within the community and between state and nonstate providers.

Given these particular challenges in mental health services delivery, qualitative research on mental health needs and services gaps in Georgia is particularly useful. The new national health care strategy emphasizes both strengthening state services and increasing privatization of services [22]. Understanding some of the reasons children in Georgia take to the streets, as well as the types of mental and behavioral health problems they experience once on the street, could significantly contribute to ongoing reforms, as well as to programmatic efforts aimed at addressing the needs of this vulnerable group. This paper describes the results of two qualitative studies conducted with street and institutionalized youth in and around Tbilisi, Georgia between 2005 and 2006. Both studies were designed to investigate local knowledge and perceptions, as well as to elicit psychosocial problems and their perceived causes in order to inform local programming. 

## 2. Study Setting

 The two studies were conducted in collaboration with Save the Children-Georgia to inform programming within their Rebuilding Lives Project, with funding from USAID Displaced Children's and Orphan Fund (DCOF) in and around Tbilisi and Gori, two urban centers. The first qualitative study was conducted in July of 2005 and focused on examining the causes of children living in the street as well as forms of healthy functioning. Children were interviewed at two centers for street children in Tbilisi and one center in Gori. These centers for street children primarily service children who return to their families at night, offering services such as daytime educational activities, meals, sports and recreation, and assistance with schoolwork. The second qualitative study was conducted in May-June of 2006 with the goal of gathering data on the problems experienced by street children and those living in orphanages. Locations for this second study included three orphanages (one in Tbilisi and two in nearby villages), and three centers for street children (two in Tbilisi and one in a small city south of Tbilisi). Orphanages in Georgia are for those children without parents, or whose parents have sent them there due to hardship (e.g., poverty, too many children). 

## 3. Method

### 3.1. Overall Study Method

 For both studies, two qualitative interview methods were used to collect data from local informants: Free Listing (FL) and Key Informant (KI) interviewing. This combination of interview techniques has been used previously to explore youth perceptions and experiences (e.g., [23–25]). Local staff hired by Save the Children received two days of training in qualitative interviewing. Training emphasized the use of nonleading, open-ended questions and probes to obtain in-depth data grounded in interviewees' own experiences. An additional day of training in specific KI interviewing techniques occurred prior to these interviews. All training was conducted in English with professional translators. All interview questions were translated collaboratively by professional translators and the local interviewers into Georgian prior to interviewing, with emphasis placed on simplicity of language for the younger children. Interviewers worked in pairs, with one conducting the interview and the other writing down (i.e., recording) the interview. After the interview was complete, the interviewers reviewed their notes together, and then sat with a translator while he/she translated the interview into English. 

 For both studies, Free Listing (FL) was conducted first to generate initial lists of perceived causes or problems. FL was done with a convenience sample of respondents, both children and adults (see below for details of each study). FL respondents also generated names of people from the local community that they believed were knowledgeable about the problems they mentioned. Topics or issues for KI interviews were selected from the resulting FL data based on response frequency, as well as the interests and resources of the local partners to address these problems. In KI interviews, identified persons are interviewed in depth to gain as much local insight as possible about the chosen KI topics. Interviewers asked KIs specific questions (see below for KI questions used in each study) and followed up with probing questions. KIs were also asked to recommend other people in the community who were knowledgeable about a particular cause or problem of interest. Most KIs were identified as being knowledgeable about more than one of the problem categories and were able to provide information on more than one topic.

 For both studies, domain analysis techniques were used to explore the data. This analysis approach consists of identifying cover terms (a term that describes a group of related concepts) and included terms (items that fall under a cover term). Patterns of cover and included terms that emerged from the interviews with one informant were compared with those from others. All analyses were done collaboratively by the research team and the local interviewers. Additional study-specific methods are detailed below, along with a general summary of results per study. The discussion section integrates both studies.

### 3.2. Study 1: Methods and Results

 Study 1 had three separate sites, which were all centers for street children: Sparrows and Rainbow Centers in Tbilisi and Biliki Center in Gori.  Table 1 shows the number of respondents for both the FL and KI Interviews. For KIs, there is a notation of how many were interviewed twice. The research team attempted to interview all KIs a second time to clarify information and/or gather additional information. A total of 10 interviewers assisted with this study. Three trainees were local graduate students in the social sciences; seven trainees were employees of local partner organizations providing assistance to street children. 

#### 3.2.1. Free List Interviews

 In the FL interviews, a total of 59 children aged 8–17 who received assistance from one of the three centers were asked four different questions, successively

1. What are the causes of children going to the street?
2. What does a happy child look like?
3. What does a happy family look like?
4. What does a happy community look like?

The questions were stated in an open-ended fashion to elicit a wide range of responses. Question 1 was written to better understand causes, while Questions 2–4 were designed to examine functioning [26]. Table 2 shows FL responses to Question 1 reported as direct translations of the Georgian phrases given by respondents. Review and analysis of responses to Question 1 (“What are the causes of children going to the street”) yielded two main categories of causes, namely (1) causes related to family issues that push children into the streets and (2) causes related to circumstances in the street that tend to keep children there. Family issues included poverty and unemployment, domestic violence, substance abuse, poor family relations, lack of attention, and changes in the makeup of the family (such as a new partner of the parent). Issues related to children remaining on the streets included the formation of friendships, discovery of ways to make money (such as begging or stealing), and the development of “habits” related to street-life, such as becoming accustomed to hanging around certain places. 

 The three function-related questions (Questions 2–4) produced a thorough description of a healthy child, family, and community. The overlap across these areas was high, with many children naming lack of fighting or quarreling, kindness to others, taking care of each other, happiness, respectfulness, and loving and caring as important characteristics of a well functioning child, family, and community. In addition, children considered all three categories (child, family, and community) to be well-functioning when accompanied by good economic conditions (e.g., employment for family members, food, toys).

#### 3.2.2. Key Informant Interviews

 Based on the findings from FL interviews and discussions with Save the Children-Georgia staff, five topics were identified for further investigation with KI interviews. Again, these were chosen based on response frequency in the FL interviews, as well as on program interests and resources for programming for Save the Children-Georgia. The five topics were

1. Fighting/Quarreling
2. Beating
3. Alcohol and/or Drug Use/Abuse
4. Relationships of Friends
5. Family Relationships

Table 3 shows the most frequent responses from KIs across all three centers, reported as direct translations of the Georgian phrases given by respondents.

Responses to questions about the topics of fighting/quarreling and beating indicated that domestic violence between parents and parent-to-child violence encouraged children to spend time on the street. Family relationships also clearly influenced a child's decision to live on the street, with KIs commonly noting a “lack of friendly relationships between parent and child” as a cause of going to the street. In addition, KIs stated that parents may force a child to beg in the street and that children may leave home to avoid this task. 

 KIs described alcohol and drug abuse as both a cause of children going to the street and an outcome of children living on the street. As a cause of going to the street, several KIs explained that alcohol and drug abuse by parents push children to the street. This substance abuse is often accompanied by violence in the home. In addition, some KIs said that children are embarrassed by their parents' addiction or the children themselves have become addicted and are forced to leave the home. KIs also stated that many children who spend time on the street become involved in drug and/or alcohol use.

 Although some KIs discussed the role of friendships (e.g., the “charm of socializing”) as among the causes of children going to the street, the majority spoke of the influence of friendships once children were spending time or living on the street. Several KIs described in depth how friendships developed on the street quickly become a child's family system, one that is “better” than their biological family. For example, many KIs explained how children living on the street help each other and share resources equally. Some of the KIs specifically described these friendships of children on the street as “stronger than other friendships.”

### 3.3. Study 2: Methods and Results

 Study 2 had six separate sites: (1) Three centers for street children (two in Tbilisi and one in Rustavi) and (2) three orphanages (one in Tbilisi, two in neighboring towns 35–45 minutes away).  Table 4 shows the number of respondents for both the FL and KI Interviews. For KIs, there is a notation of how many were interviewed twice. A total of 12 interviewers assisted with this study. Six of these interviewers had also participated in Study 1; the other six interviewers were employees of the Ministry of Education. 

#### 3.3.1. Free List Interviews

 In the FL interviews, participants were children aged 6–18 who were asked one primary question: “What are the problems of children who spend time on the street/live in orphanages?” 

Tables 5 and 6 show the most frequently mentioned problems given in the FL interviews across the three orphanages, and the three centers, respectively. The problems are reported as direct translations of the Georgian phrases given by respondents. The analysis, which was carried out with local interviewers, yielded two types of responses within each of the six sites: (1) reasons why children come to be in an orphanage or spend time on the street and (2) current problems of these children. It should be noted that these two categories of responses are not mutually exclusive and show some overlap.

 The problems that led to coming to an orphanage, as identified by children living in orphanages, included poor financial situations of families, poor relationships with parents, not having parents, having divorced parents, parents who are not able to leave their children alone at home, parents abusing alcohol, or mothers neglecting children. The issues listed by children at the centers that led them to the streets included parents forcing them to the street, not having parents, poor financial situations of families, parents abusing alcohol, poor relationships with parents, running away from home, and unemployed parents. Significant overlap of identified problems was displayed across orphanages and centers. There was a clear recognition of problems relating to parents and families, including interpersonal, financial, material, drug-related, and neglect problems, as well as simply a lack of parents. 

 The second response type was described as current problems of children in the orphanages and centers. Across the three orphanages, problems included difficulties with studies, problems with teachers and/or directors, being blamed for things or treated differently (or “differentiation”), fighting or aggression, and missing home/parents or wanting to go home. Differentiation is a term that is being applied to cover several named problems, such as “being treated differently,” “being blamed for things,” and “stigma.” Across the three centers, problems included begging, stealing, toxicomania/drug use, lack of parental warmth, hunger, wanting to be free, having no friends, feeling ashamed, misbehavior, and health problems. Toxicomania primarily refers to glue sniffing but may also include other forms of drug use such as cigarette and alcohol use. One of the clearest distinctions between the two groups of children was that children at centers listed a number of problems that orphanage children most likely do not have the freedom to engage in, such as begging, stealing, and toxicomania/drug use. The orphanage children spoke more consistently about missing home and/or their parents and wanting to be with them than did the center children. Further, the center children were more likely to talk about their parents drinking alcohol or beating them, whereas orphanage children would rarely speak negatively about their parents, and many said they would rather be at home “no matter what.”

#### 3.3.2. Key Informant Interviews

 KIs were identified by FL respondents and included children, siblings, mothers, teachers, center directors, and other staff. The following KI interview topics were chosen for Study 2 based on assessments of responses from the FL interviews across the different settings, as well as on consultations with Save the Children-Georgia.

 Orphanages:
 (a) missing parents,
 (b) relationships with parents,
 (c) differentiation.
 Centers:
 (a) lack of parental warmth,
 (b) relationships with parents,
 (c) differentiation,
 (d) toxicomania/drug use.

Tables 7 and 8 list the KI responses for the selected topics for orphanages and centers, respectively. There was significant overlap between responses to the two topics of “missing parents” and “relationships with parents.” KIs described relationships between orphanage children and their parents in both negative (e.g., fighting, scolding, alcoholic, forcing to beg) and positive (e.g., warmth from parents) terms. However, overwhelmingly, KIs described orphanage children as longing for parental warmth, wanting to see their parents, and wanting to go home with them. Many of these children did in fact still have parents and were sent to the orphanage for various reasons such as a lack of money or parents having too many children. Children who miss their parents were described as showing both internalizing symptoms (e.g., sadness/crying, being alone, worrying, being withdrawn) and externalizing symptoms (e.g., aggression, not studying, irritability). 

 The topic of differentiation emerged from KI interviews as a significant problem for children in orphanages. KIs noted that these children are treated differently from other children and mostly in a negative sense. For example, KIs frequently mentioned that children in orphanages must deal with other children not wanting to be friends with them, teachers grading them differently, and being blamed for things they did not do. Being treated differently and blamed for things had similar effects on children across the orphanages including internalizing symptoms (e.g., crying, feeling scared, downtrodden, hurt, closed, sad, looking miserable) and externalizing symptoms (e.g., justifying themselves, getting angry, aggressive).

 There was a high degree of similarity in responses given by KIs about children in centers when asked about “lack of parental warmth” and “relationships with parents.” KIs described the behaviors of parents as: beating children, forcing children to beg, displaying a lack of warmth and attention, showing neglect, displaying little love, and kicking children out of the home. The most common cause provided for these behaviors was alcohol abuse. The effects of poor relationships or a lack of warmth on children were described as feeling sad, broken-hearted, downtrodden, and oppressed, as well as crying, being closed off to others, craving affection, behaving aggressively, and going to the street. 

 Differentiation was described as others having no relation to them, making fun of them, treating them badly, and having pity on them. Symptoms included crying, trying to justify themselves, getting angry, feeling oppressed, nervous, closed off, sad, lonely, not playing with kids, and becoming aggressive (particularly to defend themselves when wronged or blamed). The primary cause listed for this differentiation/stigma was that the children do not have parents.

 Toxicomania or glue-sniffing was described as a problem resulting from children not having role models to demonstrate better behaviors and being surrounded by others who use drugs. A typical addiction pattern and symptoms were described, where once a child sniffs glue he/she cannot stop and tries to get money on the street to buy more glue. Accompanying signs and symptoms included difficulty with concentration, aggressive behavior, hallucinations and forgetting, poor hygiene, and interpersonal isolation. 

## 4. Discussion

 Two qualitative studies were conducted with children and adults at orphanages and centers for street children in Georgia to identify: Study 1—the causes of children going to the streets and indicators of healthy functioning and Study 2—problems of street children and children in orphanages. There was an initial interest in focusing on causes in Study 1 so that the “root of the problem” might be addressed in local programming. Results from the Free List interviews and in-depth, Key Informant interviews in Study 1 demonstrated that the causes of children taking to the street fall under two broad, interrelated categories: (1) “push” factors such as poverty, family problems, domestic violence, and alcohol/drug abuse and (2) “pull” factors such as the formation of friendships, economic self-reliance, and the social structure of street life. 

The variety of forms of family dysfunction, such as domestic violence, being an orphan, and parental substance abuse that led to or “pushed” children to the streets or to an orphanage was a key finding and is supported by the literature. Family dysfunction has been reported across a number of varied settings for both children on the street [27, 28] and in orphanages [29]. Similar to these study results, other researchers have also found family abuse and violence to be a cause of going to the streets [2, 28]. The effect of unhealthy social functioning within families is significant in children's decisions to leave the home [30, 31]. Poor family relationships and a lack of social support have been found to be determinants of children going to the streets [32, 33], while positive relationships with family have been linked with feelings of satisfaction among street children [1]. Families that “do not argue” and have “no conflict at home” were the most frequent responses in the FL and KI interviews with children in Tbilisi regarding what a happy family looks like. The results also clearly show that substance abuse by caregivers is an instigator of going to the streets, which has been found elsewhere as well [8, 9]. 

 Results from Study 1 highlighted familial and cultural dynamics within the “push” factors that are challenging to address and fell out of the scope of Save the Children-Georgia programming at that time. Even if these factors could be addressed, there remained “pull” factors that kept children on the street. The research team thus decided to shift to a focus on learning about problems so that youth could be supported in developing healthy coping and functioning regardless of the cause(s) or where they were (i.e., living on the street, spending time on the street, or in an institution). 

 Study 2 focused on problems of children on the street and those within orphanages. Results indicated a wide range of both internalizing and externalizing symptoms across both settings. Some of the primary symptoms described included internalizing symptoms (e.g., sadness/crying, being alone, worrying, being withdrawn) and externalizing symptoms (e.g., aggression, not studying, irritability). Research shows that street children are more prone to emotional problems such as anxiety [1], depression, and emotional difficulties [29]. It is possible that these problems result from some of the family problems discussed above such as neglect and abuse. For example, it has been reported that feelings of hopelessness increase with violence and abuse [2].

Another salient concern expressed by children and KIs was differentiation, or the sense that street children are treated differently from home-based children by their community. Differential treatment and stigma most likely exacerbate emotional and behavioral problems. The emotional and behavioral sequelae many of these children experience indicate the need for mental health and psychosocial programming for these populations. Early intervention can help stop cyclical problems within families (e.g, violence, substance abuse) and can also prevent problems in adulthood (e.g., unhealthy relationships). 

 Both studies indicated that there are “push” and “pull” factors for children spending time on the street. There were also many similarities in problems expressed by children from the street and from orphanages, such as unhealthy family relationships, parental alcohol abuse, and neglect or lack of parents. Interpersonal problems, such as feeling neglect, and a lack of parental warmth and social support, were also expressed by both groups. However, a few differences should also be noted. 

 One striking difference was that children in orphanages generally wished for and missed their parents, something that was generally not the case for children from the street. This may indicate the need for programs focused on attachment and building healthy relationships that meet some of a child's needs in the absence of a parent. Another difference between settings was higher reporting of externalizing symptoms for children from the street. These children, who spend more time on the streets and have greater freedom to move around, indicated higher levels of delinquent behaviors such as sniffing glue, stealing, and begging. These behaviors were not reported as often by children in orphanages. This may be understood as greater access to the outside world for children in centers compared to children in orphanages. Socially delinquent behaviors like committing crimes or substance abuse lead to, and are reflective of, a different range of social, rather than emotional, problems. Other studies have similarly found that street children are at increased risk for antisocial behavior, including sex work, unhealthy sexual activity [3], substance abuse, habitual lying, and violence [27]. Study findings, along with the existing literature, suggest that programs for street children should involve positive activities that would compete with these negative behaviors. As an example, a child who is spending time playing soccer will be less able to engage in a harmful behavior like smoking. 

 Less prominent but also identified in study findings was the role of peer social systems and friendships for children spending time on the streets. These social systems were found to be an integral part of surviving and remaining on the streets. Street life involves its own social structure and system, which can lead to both intergroup aggression and cooperation, as well as emotional attachment [34]. This was a phenomenon that was not mentioned in relation to socialization within the orphanages. Finally, KIs also noted that both orphaned and street children worried about stigmatization for being different or spending time without families or on the streets. The effect of stigmatization may be amplified for street children, as stigmatization affects access to and utilization of health services [35].

## 5. Conclusion

 The data collected in this study represent multiple perspectives, from street and institutionalized children themselves to community members that work with these children. The data collected illuminate the experiences of these youth and allow for the identification and understanding of constructs, behaviors, and problems relevant to this population. The study's methodology allowed for the elicitation of local terminology by preserving participants' own wording and also allowed for analysis to be performed in the original language by local interviewers. This study is one of the first to be conducted on street children in Georgia and provides specific information that can be used to inform local health initiatives for this population. 

 Findings from this study have a number of implications for interventions with street children, both in Georgia and in other settings. Many programs for street children in Georgia focus on social problems and take the form of centers or group shelters which house, educate, and help reintegrate street children back into homes or other residential settings. These programs also train children for employment opportunities. Future research should focus on conducting impact evaluations of these programs to identify what types of problems they help to decrease and what problems may not be effectively addressed by them. In addition to these types of social programs, the two studies presented here suggest a need for interventions that directly address specific social, emotional, and behavioral problems. 

 This study is a critical first step to gaining information from the perspectives of the very children and families we seek to help—those on the street or in institutions. It is evident that these children are at risk for a wide range of emotional and behavioral problems, many of which lead to problems in adulthood. Surely the economic and social problems that these children face are indicative of broader societal problems, which in the case of Georgia, reflects the country's ongoing political and economic transition. Researchers and practitioners should begin to implement and evaluate different programs that may help with the symptoms and problems reported here. 

## Figures and Tables

**Table 1:** Numbers of study 1 respondents, across sites.

	Tbilisi: two centers	Gori: one center
Free list respondents	43	16
Key informants	17 (10 twice)	12 (6 twice)

**Table 2:** Study 1 free list responses, causes of children going to the street, Tbilisi and Gori combined, (*N* = 59).

Q1. What are the causes of children going to the street?	Number reporting
They can't afford/economic hardship	49
Because of disagreement at home/parents fight	20
Are orphans	22
Parents are drunkards	20
Lack care or attention/lack of guardian	18
Parents force them to make money	15
They have no shelter (homeless)	12
Beat (parents)	11
Were thrown out of (*⋯*)	9
When they quarrel with children	9
Freedom	6
Are afraid of coming home	5
Parents can't understand children	5
Interest in street	5
Pride	5
Have sick/ill parents (beat them)	4
They have no patron	4
Meet friends in order not to be alone in the street	4
Street life becomes a custom/habit	4
For stealing	4
Because of punishment at home (the street attracted them)	4
Beggary	4

*Reported responses with > 3 respondents.

**Table 3:** Key informant responses, across all centers, (*N* = 29).

KI topics	Themes	Responses
Fighting	Description	Between mother/father and parent/child
		Threatening, beating, belittling each other

	Causes	Parents punish without reason
		Bad relationships
		Mother or father drinking alcohol
		Toxicomania or other drug use
		Child not earn enough money for family
		Child misbehaving
		Poor families
		Parent with psychological problem
		When mother is a whore

	Effects	Negatively affects child: children learn to fight

Beating	Description	Between parents, parent/child and siblings
		Group beating child if he/she causes problems on street

	Causes	Parents do not like child's behavior
		Way to get child to bring more money home
		Child stealing
		Parent does not know how to treat a child
		Parent uses drugs/alcohol
		Unstable family
		Father being upset with someone else
		Lover
		Economic need

	Effects	Child humiliated
		Child going to street

Alcohol/drug abuse	Description	Individual gets addicted; physiologic dependence

	Causes: family	Used to get rid of problems
		To be in good mood
		Lack of attention from parents
		To forget worries
		Being ashamed or tired of having addicted parents
		Unemployment of parents
		Conflict in families
		Child imitating parents

	Causes: peer relationships	Imitating friends
		To be in with a circle of friends
		Trying to prove to friends they are cool
		Interest/curiosity
		Ability to use drugs freely

	Effects	Aggression in child
		Child using
		Parents forcing child to bring home money

Family relationships	Reason children go to the street	Poor family relationships
		Constant conflict
		Beating between parents
		Parent beating a child
		Child receiving no attention
		Parents thinking only of their own happiness
		Parent prostituting

	Causes of poor family relations	Economic vulnerability
		Unemployment
		Drug/alcohol addicted parents
		Volatile parents
		Parents who ignore children
		Parent who has a lover
		Parent who sells the child or forces them to get money

Friendships	Description	Those with common problems
		Make decisions together/collaborate
		Care/help each other
		Devoted
		Share things equally
		Attracted to common adventures
		Stable/will not betray you

	Characteristics that encourage children to the street	Ability to get compassion
		Remedy for child's loneliness
		Interest in children who live on street
		Wanting to be cool

	Effects	Child stealing to help a friend
		Not caring for parents because of friends
		Forced to steal
		Smoking cigarettes
		Drink/using drugs
		Being with a bad group of friends

**Table 4:** Numbers of study 2 respondents, across sites.

	Rainbow	Sparrows	Rustavi	Kojori	Dighomi	Martkopi
	(center)	(center)	(center)	(orphanage)	(orphanage)	(orphanage)
Free list respondents	11	12	15	18	16	17
Key informants	11 (7 twice)	12 (6 twice)	14 (9 twice)	9 (4 twice)	9 (5 twice)	3 (1 twice)

**Table 5:** Study 2 free list responses, problems of children in orphanages, all orphanages (*N* = 38).

Q. What are the problems of children living in orphanages?	Number reporting
End up in the orphanage because of the financial situation/are poor	31
Want home/miss home	19
Need warmth from the mother/lack warmth/neglect	18
Some have no mother, some have no father, some have no parents at all/orphan	15
Problems with studies, or studying	15
Some do not like to be here/can't get along with regime in orphanage	13
Having problems with school teachers and the director at school	11
Some teachers are kind, some are strict	10
Problems between parents/divorce	9
They put blame on children for everything/ differentiation	9
Children fight with each other, they are treated badly	7
I am sorry for the children in the streets	7
Have no clothes	6
When someone dies, I cry	6
Children's letters do not reach the president	6
Children are aggressive	5
Flipped hand at life (don't expect anything good from life and don't try to change anything)	4
Wishes can't be fulfilled in an orphanage	4
Director was stealing everything	4
Problem of the relationship with parents	4

**Table 6:** Study 2 free list responses, problems of children on the street, all centers (*N* = 51).

Q. What are the problems of children who spend time on the street?	Number reporting
Begging	26
Are needy/poor	25
Don't have parents, or relatives/orphan	22
Sniff glue/toxic mania	21
Don't have a house	19
Have a desire to steal to satisfy their wishes	17
Kid wants to be free	14
Hunger	14
Parents force them to be in the street	11
Misbehave	9
Lack the warmth/attention of parents	9
Beating	8
Cannot get along with parents/relationship problems	8
Don't have friends/laughed at	7
Study problems	6
Problems of clothes	6
Smoking cigarettes	6
Alcoholic parent	5
When parents are far away	5
Are ashamed of begging	4
Leave home stealthily (run away without permission)	4
Health problems	4
Parents make them beg	4

**Table:** (a) Missing parents

Sign/symptom	Number reporting
Cry	14
Sad faces	11
Always misses parents	9
Aggressive	5
Worried	5
Always expecting	4
Teary eyes	4
Want to be at their homes/misses home	4

**Table:** (b) Relationships with parents

Sign/symptom	Number reporting
Mother says taking them home— happy	4
Meet parents—joy	4
Parents can't take them—sad	2
Mother beating me—escaping	2
Playing with parents—feel good	2
When sees mother, forgets how it hurt	2

**Table:** (c) Differentiation

Description	Number reporting
Kids blamed and labeled	7
Because from orphanage	4
Teachers and guards steal things and blame it on us/blamed for stealing	4
Different grading	4
They treat us well	4

Sign/symptom	

Cry	6
Justifies him/herself	4
Get angry	4
Aggressive	3
Keep to themselves	3
Scared	3
Nervous	2
Orphanage kids stick together	2
Sad	2
Verbally offend their family members	2
Look miserable	2
Blushes	2

**Table:** (a) Toxicomania

Sign/symptom	Number reporting
Have hallucinations	10
Aggressive	10
Forget their problems	9
Wear dirty clothes	9
Cannot think/mentally, their mind is not working	8
Imitate	8
Sick/sick lungs/fall ill	8
Steal	7
Can't give it up/addicted	7
Smell glue strongly	7
Inadequate behavior	6
May violate others physically/beat others	6
Killed someone accidentally/might kill someone	5
Begging	5
Have red eyes, eyes are wet, messed up, widened	5
Sniff/get high	5
Cannot stay still	4
Don't think/care of anything else	4
Create another world for themselves	4
Like drunk people	4
Burst into laughter	4

**Table:** (b) Differentiation

Description	Number reporting
Others have no close relations with them	9
Others make fun of them/talk about them	8
Not made friends with	5
Others who have experienced hardship feel pity for them	4
Others treat them badly	3
Teacher/others pays less attention to them	3
Stigma in reference to these kids/labeling	3

Sign/symptom	

Cry	11
Try to justify themselves	6
Get angry	6
Oppressed	5
Heart aches/heart broken	4
Nervous	4
Closed and won't open/don't trust	4
Sad	4
Don't enjoy talking/playing with kids	4
Alone/lonely	4

**Table:** (c) Lack of parental warmth

Sign/symptom	Number reporting
Sad	9
Aggressive	8
Closed	6
Cry	6
Sorrowful	5
Rude	5
Oppressed	5
Go away from home/to streets	5
Does not communicate with anyone	5
Do not make friends with others	4
Eyes are sad	4
In bad mood/not happy	4
Are beggars	4
Needs caressing	4
Downtrodden	4
Don't want to play	4

**Table:** (d) Relationship between parents and kids

Description	Number reporting
Parent forces the kid to beg	13
Parent beats the kid	12
Parent doesn't love her kid	6
Some have bad/tense relationships	4
Some have good relationships	4
Parent wants to let the kid in	4
Parent fights with kid	3
Child fights with his/her mother	3
Parents didn't want to have this kid	3

Sign/symptom	

Kid is strict (if parent is strict)	4
Cry	4
Child revenges against family	4
Aggressive	4
Children feel bad	3
Smoke cigarettes	3
Sad	3

## References

[1] Mathiti V (2006). The quality of life of street children accommodated at three shelters in Pretoria: an exploratory study. *Early Child Development and Care*.

[2] Duyan V (2005). Relationships between the sociodemographic and family characteristics, street life experiences and the hopelessness of street children. *Childhood*.

[3] Goodwin R, Kozlova A, Nizharadze G, Polyakova G (2004). HIV/AIDS among adolescents in Eastern Europe: knowledge of HIV/AIDS, social representations of risk and sexual activity among school children and homeless adolescents in Russia, Georgia and the Ukraine. *Journal of Health Psychology*.

[4] Guernina Z (2004). The sexual and mental health problems of street children: a transcultural preventative approach in counselling psychology. *Counselling Psychology Quarterly*.

[5] Seth R, Kotwal A, Ganguly KK (2005). Street and working children of Delhi, India, misusing toluene: an ethnographic exploration. *Substance Use and Misuse*.

[6] Densley MK, Joss DM (2000). Street children: causes, consequences, and innovative treatment approaches. *Work*.

[7] Beyene Y, Berhane Y (1997). Characteristics of street children in Nazareth, Ethiopia. *East African Medical Journal*.

[8] Pinto JA, Ruff AJ, Paiva JV (1994). HIV risk behavior and medical status of underprivileged youths in Belo Horizonte, Brazil. *Journal of Adolescent Health*.

[9] Wright JD, Kaminsky D, Wittig M (1993). Health and social conditions of street children in Honduras. *American Journal of Diseases of Children*.

[10] Grant R, Shapiro A, Joseph S, Goldsmith S, Rigual-Lynch L, Redlener I (2007). The health of homeless children revisited. *Advances in Pediatrics*.

[11] Ali M, Shahab S, Ushijima H, De Muynck A (2004). Street children in Pakistan: a situational analysis of social conditions and nutritional status. *Social Science and Medicine*.

[12] Panter-Brick C (2002). Street children, human rights, and public health: a critique and future directions. *Annual Review of Anthropology*.

[13] Morch J (1984). Abandoned and street children. *UNICEF, Ideas Forum*.

[14] Scanlon TJ, Tomkins A, Lynch MA, Scanlon F (1998). Street children in Latin America. *British Medical Journal*.

[15] IDMC (2010) http://www.internal-displacement.org/8025708F004CE90B/(httpCountries)/F62BE07C33DE4D19802570A7004C84A3?OpenDocument.

[16] Shelley L, Scott ER, Latta A (2007). *Organized Crime and Corruption in Georgia*.

[17] Lynch D (2004). *Engaging Eurasia’s Separatist States: Unresolved Conflicts and de Facto 
States*.

[18] MacPhee CR (2001). Economic education and government reform in the Republic of Georgia. *Journal of Economic Education*.

[19] MacFarlane N, Minear L, Shenfield S (1996). *Armed Conflict in Georgia: A Case Study in Humanitarian Action and Peacekeeping*.

[20] Sharashidze M, Naneishvili G, Silagadze T, Begiashvili A, Sulaberidze B, Beria Z (2004). Georgia mental health country profile. *International Review of Psychiatry*.

[21] Georgian Mental Health Coalition (2009). *Georgian Mental Health Policy Draft*.

[22] Ministry of Labour, Health and Social Affairs of Georgia (2011). *Georgia-National Health Care Strategy 2011–2015, Access to Quality Health Care*.

[23] Bolton P (2001). Local perceptions of the mental health effects of the Rwandan genocide. *Journal of Nervous and Mental Disease*.

[24] Murray LK, Haworth A, Semrau K (2006). Violence and abuse among HIV-infected women and their children in Zambia: a qualitative study. *Journal of Nervous and Mental Disease*.

[25] Betancourt TS, Speelman L, Onyango G, Bolton P (2009). A qualitative study of mental health problems among children displaced by war in Northern Uganda. *Transcultural Psychiatry*.

[26] Bolton P, Tang AM (2002). An alternative approach to cross-cultural function assessment. *Social Psychiatry and Psychiatric Epidemiology*.

[27] Olley BO (2007). Changing social and HIV/AIDS risk behaviours: effects of life skills education among urban street youths. *Vulnerable Children and Youth Studies*.

[28] Grundling J, Grundling I (2005). The concrete particulars of the everyday realities of street children. *Human Relations*.

[29] Kerfoot M, Koshyl V, Roganov O, Mikhailichenko K, Gorbova I, Pottage D (2007). The health and well-being of neglected, abused and exploited children: the Kyiv street children project. *Child Abuse and Neglect*.

[30] Thrane LE, Hoyt DR, Whitbeck LB, Yoder KA (2006). Impact of family abuse on running away, deviance, and street victimization among homeless rural and urban youth. *Child Abuse and Neglect*.

[31] Tyler KA (2006). A qualitative study of early family histories and transitions of homeless youth. *Journal of Interpersonal Violence*.

[32] Aderinto AA (2000). Social correlates and coping measures of street-children: a comparative study of street and non-street children in south-western Nigeria. *Child Abuse and Neglect*.

[33] Ferguson K (2005). Child labor and social capital in the mezzosystem: family- and community-based risk and protective factors for street-working children in Mexico. *Journal of Social Work Research and Evaluation*.

[34] Awad SS (2002). The invisible citizens roaming the city streets. *Educational Review*.

[35] Derbyshire P, Muir-Cochrane E, Fereday J, Jureidini J, Drummond A (2006). Engagement with health and social care services: perceptions of homeless young people with mental health problems. *Health and Social Care in the Community*.

